# Time-Resolved Pharmacological Studies using Automated, On-line Monitoring of Five Parallel Suspension Cultures

**DOI:** 10.1038/s41598-017-10472-1

**Published:** 2017-09-04

**Authors:** Ala A. Alhusban, Michael C. Breadmore, Nuri Gueven, Rosanne M. Guijt

**Affiliations:** 1 Australian Center of Research on Separation Science (ACROSS), School of Physical Sciences, Faculty of Science, Engineering and Technology, University of Tasmania, Private Bag 75, Hobart, Tasmania 7001 Australia; 2 School of Medicine and ACROSS, Faculty of Health Sciences, University of Tasmania, Private Bag 26, Hobart, Tasmania 7001 Australia; 3 School of Medicine, Faculty of Health Sciences, University of Tasmania, Private Bag 26, Hobart, Tasmania 7001 Australia; 4 Present Address: Department of Pharmacy, Al Zaytoonah University of Jordan, Amman, Jordan; 5 Present Address: Centre for Regional and Rural Futures, Deakin University, Geelong, Australia

## Abstract

Early stage pharmacological studies rely on *in vitro* methodologies for screening and testing compounds. Conventional assays based on endpoint measurements provide limited information because the lack in temporal resolution may not determine the pharmacological effect at its maximum. We developed an on-line, automated system for near real-time monitoring of extracellular content from five parallel suspension cultures, combining cell density measurements with a high-resolution separations every 12 minutes for 4 days. Selector and switching valves provide the fluidic control required to sample from one culture during the analysis of the previous sample from another culture, a time-saving measure that is fundamental to the throughput of the presented system. The system was applied to study the metabolic effects of the drugs rotenone, β-lapachone and clioquinol using lactate as metabolic indicator. For each drug, 96 assays were executed on the extracellular matrix at three concentrations with two controls in parallel, consuming only 5.78 mL of media from each culture over four days, less than 60 μL per analysis. The automated system provides high sample throughput, good temporal resolution and low sample consumption combined with a rugged analytical method with adequate sensitivity, providing a promising new platform for pharmacological and biotechnological studies.

## Introduction

Regulatory and ethical constraints on performing experiments on laboratory animals have directed pharmacological and toxicological studies towards cell-based based testing^1^. Cell culture experiments can be conducted with high throughput on a wide variety of cell types, with data typically derived as an end-point measurement using a plate reader. Whilst this is satisfactory for screening purposes, insight in the changes in (metabolic) biomarkers over time would provide a more profound insight in the pharmacology of the compound. To enable this, changes in nutrient, substrate and/or metabolite concentration should be determined in an automated, low maintenance manner over short intervals and correlated with cell viability. The sample consumption of such an analytical system over the duration of the cell culture experiment should be minimal, and the sterility of the cell culture should not be compromised. Current technologies available for chemical monitoring fail to meet the combination of these requirements. Sensors have good sensitivity and selectivity, but are through their specificity restricted to one analyte of interest^2^ and the signal is prone to drift and requires regular calibration. To simultaneously analyze a range of analytes, and resolve these from interferences from the media, high-resolution analytical techniques are required. Spectroscopic techniques, for example using UV^4^, FTIR^5^ or RAMAN spectroscopy^6, 7^, can provide detailed chemical information, often without the need to take a physical sample, but despite advances in the interpretation of spectra, resolving complex analyte sets with similar functional groups remains challenging. Additional, complex chemometric models need to be established and maintained through regular training sets to account for minor changes in the monitored process. Mass spectrometry can be used to analyze cell culture media, but the extensive sample clean-up often restricts its use for automated monitoring^8^. Chromatographic separations require extensive sample pre-treatment and each analysis takes >10 minutes, making it less suitable for a high throughput automated monitoring^3^. Recently, direct paper spray MS was demonstrated using a dialysis probe for monitoring extracellular glucose in adherent cell culture for 60 min, with quantitative analysis between 0.1 and 8 g/L glucose enabled through the addition of an internal standard^9^. The Zamboni group introduced monitoring of changes to the metabolome by direct MS of samples containing whole bacteria, yeast and mammalian cells^10^. For the quantitative analysis of complex analyte sets, however, a high-resolution separation may be required. Capillary Electrophoresis (CE) is a powerful and fast separation technique capable of dealing with small sample volumes^11^, and has been employed in bioprocess monitoring for broad range of analytes^12^. For example, Sandlin *et al*. employed a microfluidic CE system for *in vivo* bioprocess monitoring of extracellular amino acids through on-line coupling of the chip to a microdialysis probe^13^. Using a microfluidic perfusion approach, the secretion of glucagon from pancreatic islets of Langerhans was monitored online using an electrophoretic heterogeneous immunoassay^14^. Our group presented a sampling method for the analysis of target analytes present in the cell-free media from adhesion culture by Sequential Injection Capillary Electrophoresis (SI-CE) for monitoring lactate production^15^. Suspension cultures are more commonly used in cell culture and biotechnology. Turkia *et al*. developed a flow-through interface for a commercial CE instrument, capable of obtaining a cell-free sample from a suspension culture by on-line filtration before analysis by CE. This system was used for monitoring extracellular organic acids over several days^16^. We recently presented a sampling method for the online analysis of lactate, glucose, glutamine, and leucine/isoleucine in extracellular extracts obtained on-line from suspension cultures. This system was capable of monitoring a suspension culture of human T lymphocytes every 30 minutes over 4 days^17^.

**Figure 1:**
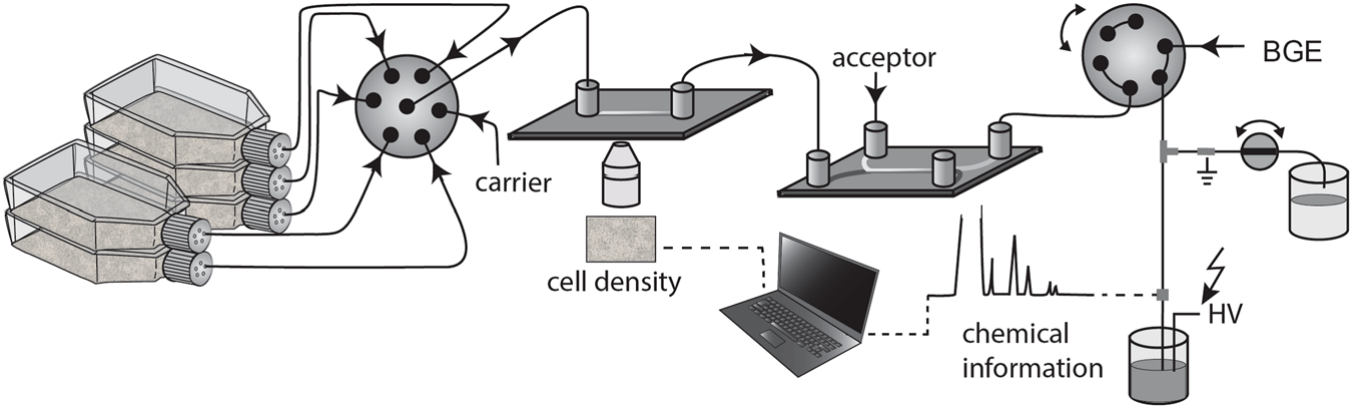
Schematic diagram of the experimental setup. Sample is sequentionally withdrawn from one of the five culture flasks, controlled by the selector valve. A carrier (5% methanol) is used to rinse to prevent cross contamination between samples. Digital imaging for cell density measurement is done using automated image analysis. The sample is then transported to a H-filter, where analytes are extracted into a cell-free solution, which is analysed by SI-CE with C4D. Fluidic isolation provided by the six port valve allows cell density measurement and extraction during the CE separation of the previous sample. With a run time of 12 minutes each flask is sampled once an hour.

Here, we present a system capable of monitoring five parallel cultures, enabling for pharmacological assays to be conducted under identical culturing conditions at several different concentrations, as illustrated in Fig. 1. To allow for monitoring parallel culture flasks, an automated fluidic system was developed that enabled switching between culture flasks for sampling. Fundamental changes to the automated liquid handling enabled increased throughput by sampling and cell density measurement during the CE separation of the previous sample. The system provided an automated, high resolution separation and cell density measurement of human T lymphocytes every 12 minutes over 4 days, giving a data point an hour from each of 5 parallel culture flasks using only 60 μL of sample per analysis. To demonstrate the applicably of the system for pharmacological studies, three different pharmaceutical compounds (rotenone, clioquinol and β-lapachone) were selected based on their potential effect on lactate production and screened at three concentrations in parallel with two control experiments. Providing time-resolved dose response data, this fully automated and flexible system can provide a new perspective to the study of biochemical and metabolic effects in cell-based pharmacological assays. Importantly, the cell density measurements allow for normalizing the data and hence for the delineation of pharmacological and toxic effects.

## Results and Discussion

Pharmacological studies are typically conducted at different drug concentrations and include one or two blanks. As biological systems are dynamic, one of the ways to minimize biological variability is to use multiple parallel flasks from the same starting culture to allow for culturing under identical conditions. To access time-resolved analytical data, each individual culture flask needs to be monitored individually. Because of the excessive infrastructure required to have separate analytical systems operating in parallel, we developed an automated, on-line sampling method that enabled the use of a single analytical system for monitoring five parallel cultures. The separations were conducted by CE combined with sample injection using a sequential injection system. We previously demonstrated SI-CE to be capable of automated and rapid high-resolution separations of small sample volumes^18, 19^. Our previous systems were able to monitor a single adhesion^15^ or suspension culture^17^. The system presented here enables simultaneous monitoring of five parallel cultures. The new fluidics here use the fluidic isolation provided by the switching valve to conduct the sampling, cell density measurement and cell removal during the electrophoretic separation of the previous sample. Compared with our previous work, this decreased the run-to-run time from 22.5 min to 12 min (Table 1), which means that when monitoring 5 parallel cultures, a data point per hour can be obtained for each individual culture.

**Table 1:** Sequence of events and volumes of BGE, sample and sample carrier solution used by the SI-CE system.

Operational step		Time	HV	Valves				Pump flowrate		Consumption		
		Time (s)	HV supply	Selector valve position	Switching valve position	3-way solenoid to	2-way solenoid position	BGE (µL.min^−1^)	Sample + carrier (µL.min^−1^)	BGE (µL)	Sample (µL)	Carrier (µL)
1	*Separation & Sampling*	452	On	2	1	Waste	Open	50	8	376.7	60.25	0
2	*Capillary flushing, sample to H-filter*	115	Off	1	1	Waste	Closed	250	90	479.2	0	172.5
3	*Equilibrate*	2	Off	1	1	Waste	Open	0	0	0	0	0
4	*Extraction (H-filter)*	60	Off	1	1	Waste	Open	50	8	50	0	8
5	*Fill T-interface with sample*	78	Off	1	2	Interface	Open	0	180	0	0	234
6	*Hydrodynamic injection*	1	Off	1	2	Interface	Closed	0	10	0	0	0.2
7	*Fill T-interface with BGE*	12	Off	1	1	Waste	Open	250	8	50	0	1.6
	*TOTAL*	720								955.9	60.25	416.3

The time-based concentration profile of a biomarker can provide insight in the effect of the administered drugs on the cells. To illustrate the potential of the system, extracellular lactate was selected as metabolic biomarker. Since different cell culture media contain a different range of compounds including amino acids, sugars, proteins, vitamins, organic and inorganic ions, the separation method developed for monitoring lactate produced by Human embryonic kidney cells (HEK293) in Dulbeccos Modified Eagles Medium (DMEM)^15^ had to be re-optimized to resolve lactate from interfering anions in the RPMI-1640 Medium used for culturing the human T lymphocyte cell line used here. BGE pH and concentration, PEI concentration, capillary coating, I.D. and length were all examined and optimized for stable performance over at least four days. The optimized conditions were as follows: 80 cm × 50 µm I.D. (10 cm to detector) fused silica capillary coated with polyelectrolytes HDMB/PSS/HDMB in combination with a BGE containing, 35 mM Tris/35 mM CHES at pH 8.9 with 0.025% PEI.

### Analytical Performance of the Multi-Flask System

**Figure 2:**
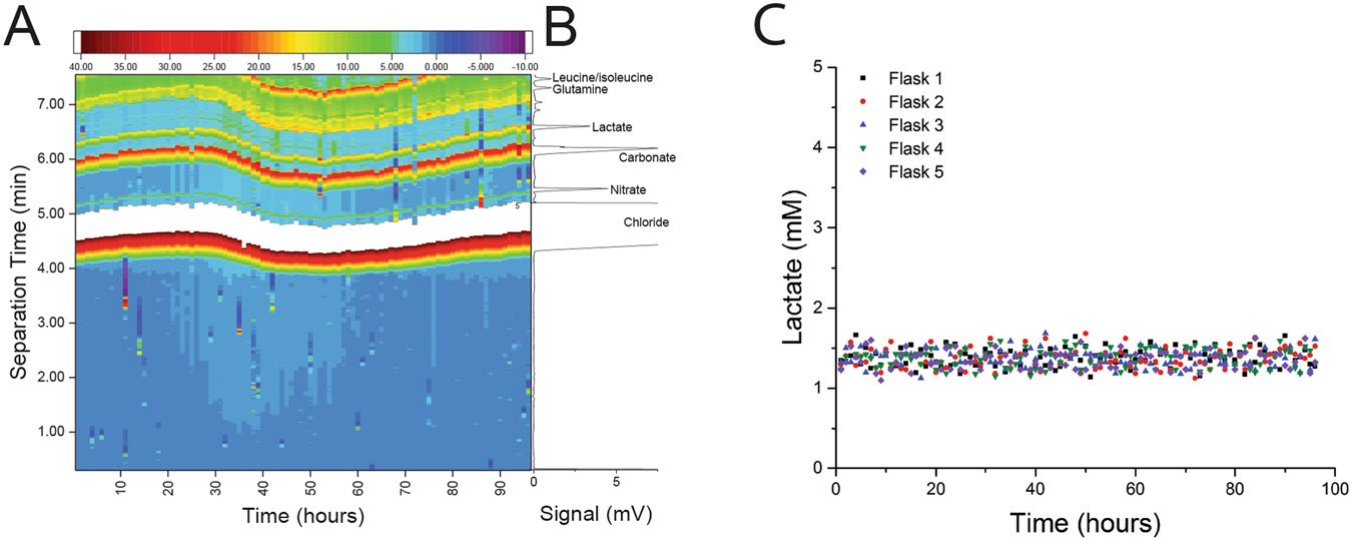
(**A**) Repeatability in the detection of analytical targets in RPMI-1640 cell culture media with 10% FCS (without cells) in one flask over continuous 4 days, (**B**) corresponding electropherogram showing chloride, nitrate, carbonate, lactate, and amino acids glutamine and leucine/isoleucine. Conditions: 80 cm × 50 μm I.D. × 350 μm O.D. fused silica capillary coated with HDMB/PSS/HDMB; BGE: 35 mM Tris/35 mM CHES, pH 8.9 with 0.025% PEI; +28 kV, C: Repeatability of lactate concentrations in RPMI-1640 cell culture media with 10% FCS (without cells) in five flasks over continuous 4 days.

Evaluation of the system and separation chemistry was performed at the intermediate level, sampling from a single flask of culture media over 4 days. The peak area for lactate was corrected using that of the chloride peak as internal standard to eliminate any variation resulting from changes in injection volume. The repeatability of the separation is depicted in Fig. 2A with the corresponding electropherogram given in Fig. 2B. The method was validated, with precision of the corrected migration times of 0.71% RSD (n = 5) and 0.26% RSD (n = 5) for interday and intraday respectively. The precision in normalized peak area was 7.88% RSD (n = 5) and 5.97% RSD (n = 5) for interday and intraday, respectively. The system was calibrated by using standards containing 0.15, 0.45, 1.35, 4.00, 8.00 and 15.00 mM lactate in RPMI-1460 media with 10% FCS, corrected for chloride as internal standard. Based on 5 injections at each concentration, the detector response was determined to linear from 0.15–15 mM (y = 28.992x + 14.735, r^2^ = 0.9931). The LOD for lactate was 9 µM with a LOQ of 17 µM (determined using S/N = 5 and S/N = 3, respectively).

To assess the ruggedness of the analysis from the five flasks, the same culture media (without cells) was distributed across five flasks. The system sampled hourly from each of these 5 flasks over 4 days. Processing of the data indicated a lactate concentration of 1.37 mM. As shown in Fig. 2C, there is an excellent correlation between the lactate concentrations in each flask (1.37 mM ± 0.12 in flask 1; 1.37 mM ± 0.12 in flask 2, 1.37 mM ± 0.11 in flask 3, 1.38 mM ± 0.12 in flask 4, 1.37 mM ± 0.11 in flask 5) (p = 0.765). The variability in lactate concentration between flasks is less than 7.65% across 4 days.

Because the drugs selected for this study were selected because of their reported effect of on extracellular lactate concentration, all data analysis reported here focuses on lactate. For glutamine and leucine/isoleucine, similar profiles are expected, demonstrating the richness of the data that can be obtained using this method. To account for the lactate present at t = 0 s, the metabolic activity in this paper is described as a change in lactate concentration (Δ_lactate_), not in absolute lactate concentration.

### Simultaneous Monitoring of Five Parallel Cell Cultures

**Figure 3:**
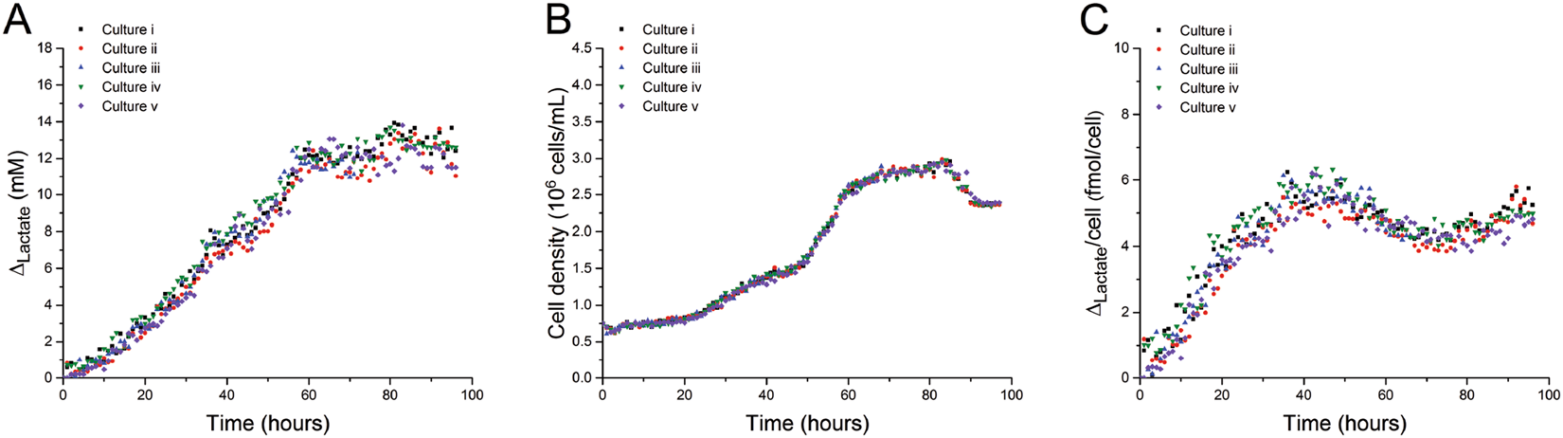
Simultaneous monitoring of 5 parallel cell cultures of Jurkat cells over 4 days. (**A**) Changes in lactate concentration determined using the conditions detailed in Fig. 2, (**B**) Changes in cell density, (**C**) Lactate concentrations standardized on cell density. No significant differences were found between the 5 parallel cultures based on the rate of lactate production per cell per time, (*p* values range 0.072–0.791), using one way ANOVA test.

Biological systems are dynamic systems where the metabolic behavior should be repeatable when the culture conditions are precisely controlled. To study the biological variability, 5 parallel cell culture flasks from a common culture of T-lymphocyte cell line (Jurkat) were cultured under identical conditions. Following the protocol described in Table 1, a cell-free sample was analyzed every 12 min; one per bioreactor per hour. It is important to note that the cells were removed without lysis, hence the sample only contained media and extracellular compounds. To correlate Δ_lactate_ with cell density over 96 hr, a total of 480 electropherograms and 480 images were analysed: 96 electropherograms and 96 images for each culture flask. Each analysis required 60.25 µL of culture media, hence 5.78 mL of media per flask was used for analysis over four days, which is less than 10% of the initial media volume. For the 5 parallel cultures Δ_lactate_ showed good similarity over the culture period over 4 days (Fig. 3A), with a combined variability of 12.05%. The cell growth, measured by the increase in cell density over time, also showed excellent similarity across the five flasks (Fig. 3B). As illustrated in Fig. 3C, the variability in the Δ_lactate_ per cell was also low. The high level of similarity obtained analyzing the data obtained for 5 cultures in parallel provided the confidence that the developed system could be applied to study the effect of pharmaceutically active compounds known to influence lactate production. Additionally, combining the variability of 12.5% in presence of cells with the 7.65% analytical variability reported in Fig. 2 provides a unique insight in the biological variability introduced by the cells. Sealing of the Tygon tubing in the coverlids with silicone was critical to keeping the cultures infection-free, with the incidence of infection/contamination less than 1 in 15 flasks.

### Pharmacological Applications

**Figure 4:**
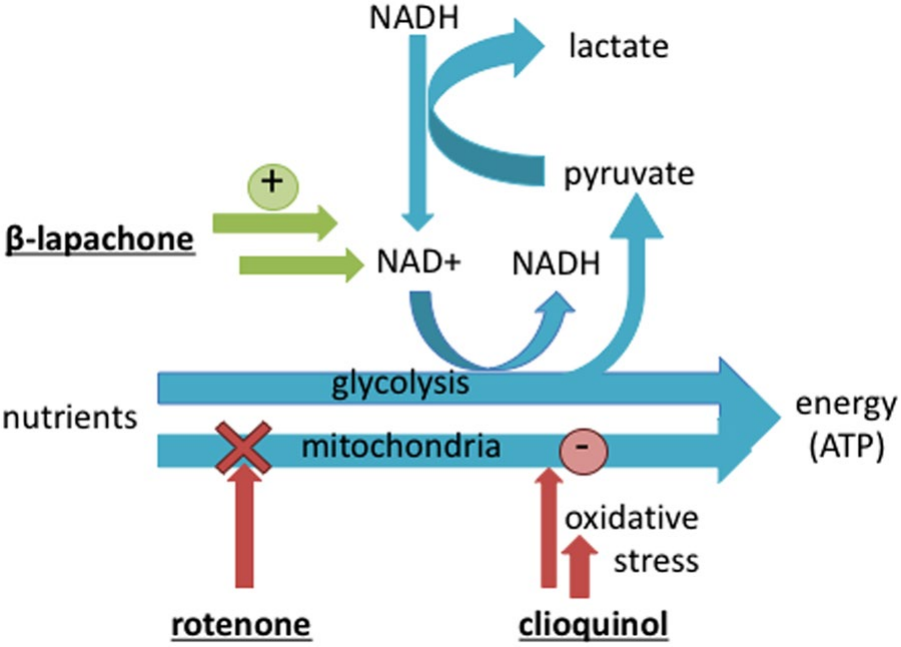
Schematic explanation of lactate production in cells because of the generation of NAD+ from NADH to sustain glycolysis. Rotenone is a mitochondrial poison, inhibiting mitochondrial function; cloquinol causes oxidative stress and as a result compromises mitochondrial function and β lapachone results an increase of NAD+, which is believed to reduce the need for lactate production.

Lactate is considered as one of the most important biomarkers of metabolism and energy status in mammalian cell culture^20^. Normally, lactate is produced from glucose and glutamine in mammalian cells as a result of their conversion into ATP^21^. Lactate levels are inversely correlated to mitochondrial function, as impaired mitochondrial function will enhance glycolysis to meet the cells energy needs. This in turn relies on the conversion of pyruvate into lactate to generate nicotinamide dinucleotide NAD+ required for glycolysis. Mitochondrial impairment has been demonstrated to increase lactate production in Jurkat cells^22, 23^, whereas, enhancing mitochondrial oxidative phosphorylation has been demonstrated to decrease lactate production in tumor cells^24^. Three drugs with reported metabolic effects were selected to demonstrate the SI-CE system for monitoring parallel suspension cultures. The way these drugs are thought to affect mitochondrial function is depicted in a simplified schematic in Fig. 4.

### A Mitochondrial Inhibitor – Rotenone

**Figure 5:**
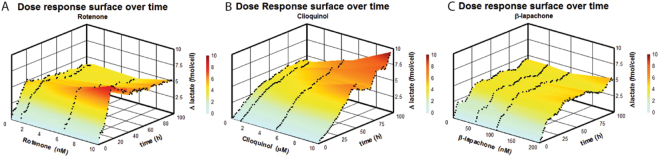
3D surface diagrams graphically illustrating the relationship between lactate production per cell and drug concentration over 96 hr for (**A**). Rotenone, (**B**). Clioquinol and (**C**). β-lapachone. The change in lactate was determined by SI-CE using the conditions described in Fig. 2 and corrected for cell density.

Rotenone is utilized as an insecticide and its toxicity is based on inhibiting mitochondrial respiration^25^. Rotenone is a high affinity mitochondrial NADH dehydrogenase (complex I) inhibitor, which at nM concentrations increases extracellular lactate levels^26^. Complex 1 is essential for mitochondrial oxidative phosphorylation^27^. Preliminary cell toxicity testing (data not shown) indicated a non-toxic concentration below 10 nM, hence it was decided to test the SI-CE system for monitoring bioreactors with 1 nM, 6 nM and 10 nM rotenone in parallel with two control experiments in the remaining two flasks. The data are summarized as Δ_lactate_ per cell in Fig. 5, with underlying experimental data given in the Supplementary Information. The non-corrected data in SI Figure 1A indicate 1 nM and 6 nM rotenone increased lactate production, whereas treatment with 10 nM rotenone decreased lactate production in comparison with the control flasks, a trend that is inconsistent with its known pharmacological behavior. However, rotenone is also known to be cytotoxic and can cause cellular death^28^. The cell density profiles (Fig. SI–1B) confirm a dose- and time-dependent reduction in cell growth compared to controls. The standardized results (Fig. SI–1C) agree with the established pharmacological behavior and demonstrate increased lactate production. This confirms our earlier conclusion that it is important to correct Δ_lactate_ for cell density^17^. The standardized lactate production is plotted against the variables rotenone concentration and time in a 3D surface plot in Fig. 5A. Treatment with 1 nM rotenone showed no measurable difference in Δ_lactate_, suggesting this concentration was below the effective inhibitory concentration. The end-point measurement at 96 hr indicates only lactate production from the 10 nM rotenone treatment was distinctly different from the control and other concentrations (p < 0.001). When considering the time-based profiles, however, we find that the rate of lactate production per cell differed across the rotenone concentrations over the first 20 hr. For the 10 nM treatment, lactate was produced at 0.44 fmole/(cell.hr) which is significantly different from the control (p = 0.002). For the flask using 6 nM rotenone, this was 0.31 fmole/(cell.hr), also significantly different from the control (p = 0.029). For the 1 nM, the measured 0.20 fmole/(cell.hr) was not significantly different from the control 0.24 fmole/(cell.hr) (p = 0.149). From Fig. 5A we can conclude that after about 20 hr, the cell metabolism switched from lactate production to lactate consumption as evidenced by the decrease and stabilization of the amount of lactate produced per cell. At this stage, the effect of rotenone is no longer noticeable on Δ_lactate_ per cell.

### A Mitochondrial Toxin – Clioquinol

Clioquinol (CQ) has been clinically used as an antibiotic and antifungal agent^29^ before it was removed from the market due to neurotoxicity that was likely related to impaired mitochondrial function^30^. CQ indirectly induces mitochondrial dysfunction by increasing oxidative stress and lipid peroxidation (Fig. 4). However, the cellular response to compensate for reduced mitochondrial ATP synthesis increases glycolysis, similar to the metabolic effect of rotenone. Therefore, CQ treatment of cells was expected to increase lactate production like rotenone. CQ was added to three parallel cultures at 1 µM, 5 µM and 10 µM, respectively, and together with two controls, these five flasks were monitored simultaneously under identical conditions. Looking at the raw data, lactate production (Fig. SI-1D) in controls and 1 µM CQ was similar (p = 0.971), and an unexpected decrease in lactate production was observed for 5 µM and 10 µM CQ. This decrease in lactate production coincided with a dose-dependent reduction in cell density compared to the control cultures (SI Fig. 1E).

Considering Δ_lactate_ per cell (Figure SI-F), a dose-dependent increase in lactate production is observed. The 3D surface visualizes the dose-dependent effect of CQ on Δ_lactate_ per cell over time (Fig. 5). The increase in lactate production indicates that CQ decreased the metabolic activity of Jurkat cells by impairing their mitochondrial function. In this case, the valuable information added by monitoring the culture rather than relying on end-point measurements is that while the lactate production rate is the same for the treated and control experiments, a correlation between the duration of the lactate producing phase and CQ concentration can be observed. This may be explained by the fact the rate at which cells change from lactate production to lactate consumption may be influenced by oxidative stress. The apparent increase in Δ_lactate_ per cell between 45 and 75 hr for the 10 µM CQ treated culture is most likely the result of increased imprecision caused by decreased reliability of the cell density measurements at the low cell density resulting from CQ toxicity (Fig. SI-1E).

### A Mitochondrial Activated Cytotoxin – β-lapachone

β-lapachone, a naphthoquinone derivative, increases the intracellular NAD+/NADH ratio by enabling the NQO1-dependent oxidation of NADH to NAD+^31^. Jeong *et al*. reported a reduction in lactate production by 1 µM β-lapachone in two hybrid cell lines^32^. The reason for this reduced lactate production is thought to be partially a consequence of increased NAD+ levels by β-lapachone, alleviating the need for NAD+ production through the conversion of pyruvate into lactate (Fig. 4). The previously reported β-lapachone concentration (1 µM) resulted in significant cytotoxicity in our cell line, with normal cell growth only observed at 0.2 µM or below. Therefore, monitoring experiments were conducted with β-lapachone at 50 nM, 100 nM and 200 nM, again combined with two parallel controls. In the raw data, the only significant difference in Δ_lactate_ was found between 0.1 µM treatment and control (p = 0.015) (Fig. SI-1G). No dose-dependent effects were observed on cell density (Fig. SI-1H). No statistically significant differences in Δ_lactate_ standardized by cell density were observed between the rate of lactate production per cell between 50 nM and control (p = 0.714) (Fig. SI-1I), between 100 nM and control (p = 0.7644) and between 200 nM and control (p = 0.145), using a one-way ANOVA test. The surface plot derived from the standardized lactate production for the different concentrations over time confirms there are no dose-related effects on Δ_lactate_ per cell for β-lapachone over the 4 days (Fig. 5C). This suggests β-lapachone does not affect Δ_lactate_ per cell. Considering the toxicity of β-lapachone, the previously reported decrease in lactate production may have been the result of a decrease in cell density rather than an actual effect on lactate production^32^. This again demonstrates the value of our system by being able to provide the data required to standardize on cell number for metabolite measurements over time.

From these tests, we can conclude that the SI-CI system for monitoring 5 parallel suspension cultures is suitable for conducting parallel, time resolved cell assays over at least 96 hr. The time-dependent profile provides deeper insight in the pharmacodynamics of the administered compound, especially when these effects vary over time. 3D surface plots provide a means to visualize the times of lowest and highest response for a specific drug and dosage. The temporal insight provided by the presented method is unique and provides a better understanding of the underlying metabolic effects.

When comparing the three surface plots, the control profiles (no drug added) vary for the three different experiments. Based on the excellent agreement documented for the five parallel cultures in Fig. 3, these differences are most likely the result of a difference in metabolic starting point between these three experiments. This again emphasizes the importance of having parallel controls analysed with the drug studies to be able to delineate pharmacological effects from effects related the metabolic starting point. Recognizing the current approach only determines extracellular lactate, further studies are required to determine intracellular effects. Based on the presented data, it appears the differences in Δ_lactate_ per cell over time may be indicative of the different pharmacology of rotenone and clioquinol.

## Conclusion

A fully automated system for monitoring extracellular media for five parallel suspension cultures was developed and applied to study the effects of three different pharmaceutically active compounds. Combining a high-resolution separation with cell density measurement, a SI-CE system was integrated with microfluidic units for cell counting and the extraction of analytes into a cell-free sample. Streamlining of the fluidic operations allowed for a run-to-run time of 12 min, enabling an analysis per hour for each of the five culture flasks. Combined with low sample consumption (60 µL/analysis) and high throughput (480 analyses in 4 days), this system provides a new approach to study time-resolved pharmacological and metabolic effects in *in-vitro* assays. The system was applied to monitoring lactate, with the interday precision of the electrophoretic mobility and peak area 1.35% RSD and 8.11% RSD respectively. A good correlation was obtained for cell density and lactate measurements across identical parallel cultures (p = 0.765). When applied to a pharmacological assay for rotenone, clioquinol and β-lapachone, concentration-dependent increases in the production of lactate was observed for rotenone and clioquinol, with their pharmacological differences reflected in changes in the rate of lactate production over time. For rotenone, a steep increase was observed over the first 20 hr, whereas for cliquinol the pharmacological effect was most noticeable over the 20–96 hr interval. This difference may have gone unnoticed when solely relying on end-point measurements. After correction for cell density, no concentration effect on lactate production could be identified for β-lapachone, suggesting earlier reports of decreased lactate production may have been the result of toxicity causing cell death.

With the target analytes defined by the separation chemistry, the proposed system provides a flexible platform, where other targets can be accommodated for changing the separation chemistry, with no or minimal changes to the hardware. Integration of the cell count and H-filter devices, as well as further miniaturization of the system would lead to increased throughput and simplify the setup, while the reduction in dead volume would further reduce sample consumption.

## Methods

### Chemicals and Reagents

All chemicals were obtained as analytical grade reagent from Sigma–Aldrich, (Sydney, Australia) and used as provided unless indicated otherwise. All solutions were prepared in Milli-Q water (Millipore, Bedford, MA, USA) and micro-filtered prior usage. 20 mM lactate, 10 mM L-glutamine, 5 mM L-leucine, 5 mM L-isoleucine, 5 mM L-arginine and 2 M chloride standard solution were prepared and stored at 8 °C. Rotenone, β-lapachone and clioquinol (Ciba chemicals, Basel, Switzerland) were all prepared at 10 mM and frozen before dilution and addition to culture media. The cationic polyelectrolyte poly(ethylenimine) (PEI) (ACROS organics, Geel, Belgium) at 0.025% was added to BGE. An 80 cm polyelectrolyte coated fused-silica capillary was prepared by flushing the capillary with hexadimethrine bromide (HDMB) and poly(sodium 4-styrene sulfonate homopolymer) (PSS) and (HDMB) to reverse the EOF. The BGE consisted of a combination of tris(hydroxymethyl) aminomethane (Tris)) and N-cyclohexyl-2-aminoethanesulfonic acid (CHES) at pH 8.9.

### Instrument Design

The SI-CE instrumentation was modified from a design developed earlier in our laboratory^15^. It consists of two peristaltic pumps (PeriWaves, CorSolutions, Ithaca, NY, USA) for sample introduction and BGE delivery. In comparison with the previous system, a 7-port selector valve (MXP-7970, Rheodyne, Oak Harbor, WA, USA) was added to enable sequential sampling from the five culture flasks. A microfluidic chamber was employed to determine the cell density, an H-filter to prevent cells and debris from clogging the injector valve. A three-way solenoid valve (360T041SHH, NResearch, West Caldwell, NJ, USA) was used to control flow of either the extracted sample or the carrier solution into the injector. A high-pressure two-position six port switching valve (MXP9900-000, Rheodyne, Oak Harbor, WA, U.S.A) was used to deliver either BGE or sample to the capillary interface. A PEEK T-piece-connector (P-727, Upchurch Scientific, OakHarbor, WA, U.S.A.) was used to interface the flow injection system with the separation capillary. A fused silica capillary (50 μm I.D.; Polymicro Technologies, Phoenix, AZ, USA) was used for separation and its inlet was positioned in the interface to eliminate any carry over, with the outlet immersed in a glass vial filled with 25 mL BGE. A 20 mm long stainless steel syringe needle was used to connect the T-interface with Teflon tubing (1.6 mm O.D., 0.5 mm I.D.) and used as a hollow electrode. A two-way solenoid valve (HP225K021, NResearch, West Caldwell, NJ, USA) was used to stop the flow between the separation capillary and the waste to perform a hydrodynamic injection. Detection was realized using a capacitively coupled contactless conductivity detection (C^4^D) (Tracedec, Istech GmbH, Strasshof, Austria) with optimized operational parameters as follows: frequency, 2x high; voltage, 18 Db; gain, 200%; off set, 000. A high-voltage power supply (4300 Emco, Sydney, NSW, Australia) with positive polarity was connected to the anode immersed in the outlet BGE vial. A NI USB-6212 data acquisition interface board (National instruments, Austin, TX, U.S.A.) using LabView v8.1 (National Instruments) was used to control the peristaltic pumps, selector and solenoid valves and power supply, and for collection and saving the data.

### Cell Density Measurement

A microscopy chamber (1 μ-slideVI^0.1^, Ibidi GmbH, Martinsried, Germany) prepared as described previously was placed on the stage of a phase contrast microscope connected to a digital camera (AM7023B Dino-Eye, New Taipei City, 241 Taiwan) to count cells by image capture. Images were automatically captured at 40x magnification every 12 minutes as programmed to correlate with sample injection into the SI-CE. Images were analyzed to determine % surface area covered by cells or aggregates using the freeware package Image J to determine the cell density. Reproducibility and validation of this procedure are discussed elsewhere^17^.

### H-Filter

An H-filter with a 40 mm long, 1 mm wide and 0.285 mm high central channel was made by soft lithography in PDMS using a dry film photoresist template (EP SUEX TDFS Sudbury, MA, USA) created using a laser printed transparency mask^17, 33^. The H-filter was employed to acquire a cell- and particulate-free solution with at least 30% of the target analytes in the acceptor stream at a flow rate of 8 μL.min^−1^. Design, fabrication, validation and extraction efficiency are described in detail in our previous work^17^.

### Instrument Operation

The SI-CE setup is schematically depicted in Fig. 1 and was operated in 7 consecutive steps. The 7-port selector valve was switched to withdraw sample from the first culture flask at the lowest flow rate to enable the image capture of the cells inside the counting device while the three-way solenoid was switched to flush the rest of previous sample to the waste.

Simultaneously, the switching valve was switched back to direct BGE towards the T-interface at low flow rate while the two-way solenoid was open, and the high voltage (HV, + 28 kV) was applied on the capillary for the electrophoretic separation of the previous sample. The selector valve was switched back to withdraw the sample carrier solution at higher flow rate to carry the sample through the sample peristaltic pump into the H-filter. At the same time, the two-way solenoid was closed and both the T-interface and capillary were filled and flushed with BGE at higher flow rate to equilibrate the separation capillary, remove any air bubbles or partial blockage and stabilize the baseline. The sample flow rate was lowered back to 8 µL.min^−1^ to enable the diffusion of target analytes away from the cells in the donor into an acceptor solution in the H-filter. In parallel, the two-way solenoid was reopened and the flow of BGE reduced to eliminate the pressure in the T-interface. The three-way solenoid was switched to transport the cell-free media into the switching valve at higher flow rate. The switching valve was switched to direct sample to the T-interface and the two-opening solenoid valve was closed for one second to hydrodynamically inject the sample plug into the separation capillary. In the final stage of this run, small amount of the carrier solution was withdrawn to initiate the sample pump for the next run. And in the meantime, the switching valve was switched to deliver BGE to clean the T-interface from the rest of the sample at a higher flow rate, and to prepare the setup to begin the next run. Events sequence, conditions and liquids volumes were cautiously optimized and are detailed in Table 1. All separations were performed at room temperature (20 °C).

### Electrophoretic Conditions

Separations were performed using fused-silica capillaries (50 µm I.D., 360 µm O.D. and 80 cm in length, L_D_ = 70 cm). Capillaries were conditioned by flushing with 1 M NaOH at 0.5 µL.min^−1^ for 10 min then Milli-Q water at the same rate for 5 min. For polyelectrolyte coating, the capillary was flushed with 1% aqueous solution of HDMB for 10 min at 0.5 µL.min^−1^, Milli-Q water at the same rate for 5 min, 1% PSS for 10 min at 0.5 µL.min^−1^, Milli-Q water at the same rate for 5 min, 1% aqueous solution of HDMB for 10 min at 0.5 µL.min^−1^ and finally with BGE for at least 30 min. The optimized BGE consisted of 35 mM of Tris (tris(hydroxymethyl)-aminomethane) and 35 mM CHES (cyclohexyl-2-aminoethanesulfonic acid) at pH 8.86. 0.025% (w/v) PEI was added for coating stabilization and selectivity alteration. Electrophoretic separations were conducted at 28 kV. A nitrogen gas line was connected to the BGE bottle to prevent change of the pH from the absorption of CO_2_.

### Cell Culture

The human T lymphocyte cell line (Jurkat ATCC^®^ TIB-152) was cultured in T75 cell culture flask (Corning^®^ 75 cm^2^ rectangular canted neck cell culture flask with vented cap) routinely at 37 °C and 5% CO_2_ in a cell culture incubator, in RPMI-1640 Medium (23.8 mM NaHCO_2_, L-glutamine without HEPES; 10% fetal calf serum; FCS, VWR, Murarrie, Australia). For the assays, 225 million cells were centrifuged at 200 g for 5 minutes at room temperature. Pellets were re-suspended gently in 200 mL culture medium and transferred into a 500 mL Erlenmeyer flask to form a total volume of 300 mL, equivalent to 7.5 × 10^5^ cells.mL^−1^. This 300 mL was distributed equally over 5 Pyrex^®^ 250 mL Erlenmeyer flasks with screw cap (4985, Fisher Scientific, USA), adding 60 mL to each. These flasks were positioned and fixed in an orbital shaking water bath (OW1412, Paton Scientific, Victor Harbor, SA, Australia) at 37 °C and at 100 rpm. Water covered entire flasks to the neck to maintain a constant temperature through culturing time. The coverlid of each flask was drilled with a 0.95 mm diameter hole to insert (0.91 mm O.D., 0.19 mm I.D) Tygon tubing (SC0001T, ISMATEC, Wertheim, Germany) for sampling. All five tubing sections were connected to five inlet ports of the selector valve at positions (no. 2 to 6). Position no.1 was connected to a carrier solution (5% methanol solution). A (0.91 mm O.D., 0.25 mm I.D) Tygon tubing (SC0002, ISMATEC, Wertheim, Germany) was connected to the selector valve outlet (position 7) and to the inlet of a counting chamber positioned on a microscope connected to a digital camera (AM7023B Dino-Eye, New Taipei City, 241 Taiwan) for image capturing for cell density determinations. The sample peristaltic pump was connected to the outlet of the counting chamber. As careful sterility guarantee is very important to ensure reproducible and accurate outcomes the culture flasks, Tygon tubing with coverlids, peristaltic pump tubing and the H-filter were autoclaved prior their use. The whole setup was fitted in a laminar flow cabinet (ESCO, Singapore) to diminish the risk of contamination. Serial dilutions with media were performed to realize the indicated concentrations of respectively clioquinol, β-lapachone and rotenone in the culture flasks, concentrations are reported as final concentrations during the experiment.

## Electronic supplementary material

Supplementary Information
