# Supplementary material for: Time-Resolved Pharmacological Studies using Automated, On-line Monitoring of Five Parallel Suspension Cultures
Source: Sci Rep. 2017 Sep 4;7:10337. doi: 10.1038/s41598-017-10472-1 (PMC5583285; doi:10.1038/s41598-017-10472-1)
Supplement: Supplementary file 1 — Supplementary Information [file 41598_2017_10472_MOESM1_ESM.pdf]

Time-Resolved Pharmacological Studies using Automated, On-line Monitoring of Five Parallel Suspension Cultures

Ala A. Alhusban Michael C. Breadmore, Nuri Gueven, Rosanne M. Guijt

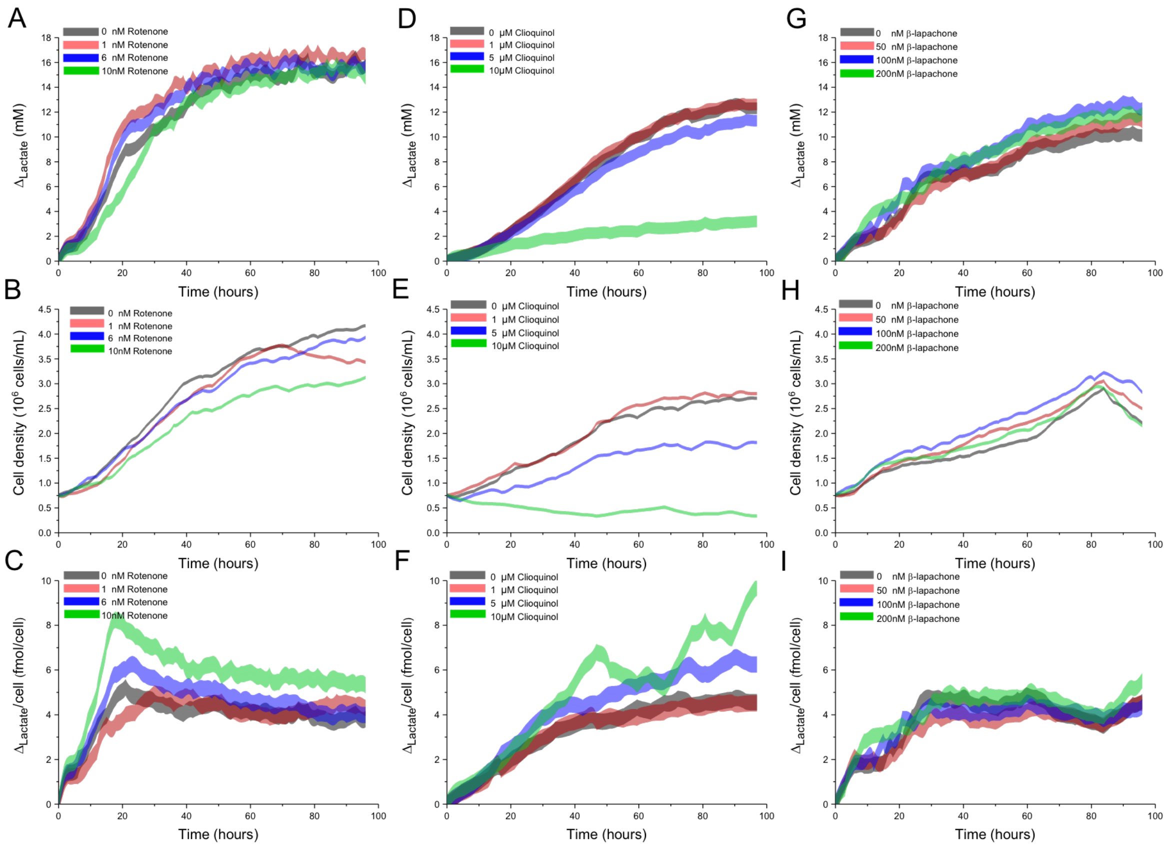

Figure SI-1 Monitoring of 5 parallel Jurkat cell cultures over 4 days in the absence or presence of **Rotenone** (A, B and C), **Clioquinol** (D, E and F) and **β-lapachone** (G, H and I) (shown as lines with SD error-bar as colored bands).

1 nM, 6 nM, 10 nM of **rotenone** was added to individual cell cultures: Changes in lactate concentration (n=5), B: Changes in cell density (n=5), C: Changes in lactate concentrations standardized on cell density (n=5). Statistical significant differences, based on the rate of lactate production per cell per time, were obtained between 10 nM treatment and control ($p < 0.001$) and 6 nM treatment and control ($p = 0.046$), whereas no significant difference between 1 nM treatment and control ($p = 0.413$).

1 µM, 5 µM, 10 µM of **clioquinol** was added to individual cell cultures D: Changes in lactate levels over time (n=5), E: Changes in cell density (n=5), F: Changes in lactate concentrations standardized on cell density (n=5), statistical significant differences, based on the rate of lactate production per cell per time were obtained between 10 µM treatment and control ($p < 0.001$) and 5 µM treatment and control whereas no significant difference was found between 1 µM and control.

50 nM, 100 nM, 200 nM of **β-lapachone** was added to individual cell cultures G: Changes in lactate levels over time (n=5), H: Changes in cell density (n=5), I: Changes in lactate concentrations standardized on cell density (n=5), no significant differences were found between treatments and control based on the rate of lactate production per cell per time, using one way ANOVA test.
